# Laser interstitial thermal therapy of a single nodule in a complex epileptic network with multiple periventricular nodular heterotopias

**DOI:** 10.3171/2024.4.FOCVID2417

**Published:** 2024-07-01

**Authors:** Sami Obaid, Albert Guillemette, Samuel Lapalme-Remis, Joseph Yuan-Mou Yang, Alexander G. Weil, Alain Bouthillier

**Affiliations:** 1 University of Montreal Hospital Center Research Center (CRCHUM), Montréal, Québec, Canada;; 2 Division of Neurosurgery, University of Montreal Hospital Center (CHUM), Montréal, Québec, Canada;; Departments of 3 Surgery and; 4 Neuroscience, University of Montreal, Montréal, Québec, Canada;; 5 Division of Neurology, University of Montreal Hospital Center (CHUM), Montréal, Québec, Canada;; 6 Neuroscience Advanced Clinical Imaging Service (NACIS), Department of Neurosurgery, The Royal Children’s Hospital, Melbourne, Victoria, Australia; and; 7 Division of Pediatric Neurosurgery, Department of Surgery, Sainte Justine Hospital, Montréal, Québec, Canada

**Keywords:** laser interstitial thermal therapy, periventricular nodular heterotopia, optic radiations, tractography, epilepsy surgery

## Abstract

Surgical management of drug-resistant epilepsy (DRE) in patients with multiple periventricular nodular heterotopias (PVNHs) is challenging. Identifying the location of seizure onset within these complex epileptic networks is difficult, and open resection carries risks of injury to surrounding functional white matter tracts such as optic radiations (ORs). The authors demonstrate tractography-assisted laser ablation of a single nodule in a patient with DRE and multiple PVNHs. Following surgery, visual fields were intact, highlighting the benefits of OR tractographic reconstruction. At 12 months postoperatively, the patient remained seizure free, suggesting the potential efficacy of targeting a single heterotopia within complex networks in well-selected cases.

The video can be found here: https://stream.cadmore.media/r10.3171/2024.4.FOCVID2417

## Transcript

Here we describe the decision-making and the surgical steps involved in laser thermal ablation of a single nodule within a complex epileptic network with multiple periventricular nodular heterotopias.

### 0:35 Introduction.

Laser interstitial thermal therapy is a minimally invasive stereotactic alternative to open resection in patients with drug-resistant epilepsy.^1^ This surgical approach causes ablation of brain lesions using laser thermal energy under real-time MR thermography.^1^ Surgical series in the past 10 years have shown its safety and efficacy for treating deep-seated lesions and lesions within or near eloquent regions.^1^ LITT has been shown particularly effective to treat epileptogenic lesions such as mesial temporal sclerosis, hypothalamic hamartomas, malformations of cortical development, and cavernomas, leading to good or excellent seizure control rates.^2–8^

The following case will illustrate the decision-making, the perioperative workup, and the operative technique, including surgical adjuncts, in a patient with drug-resistant epilepsy who underwent laser interstitial thermal therapy of a single periventricular nodular heterotopia within a complex epileptic network.

### 1:37 Clinical History.

Our patient is a 30-year-old right-handed man with a 6-year history of epilepsy. His seizure semiology consisted of focal impaired awareness seizures during which he experienced a blurred vision and a sensation of anxiety rising from the chest to the head. His seizures occurred on average 5 times per week despite being on and having tried multiple antiseizure medications.

### 2:04 Findings on Scalp Video-EEG Monitoring and MRI.

As part of presurgical evaluation, a panel of noninvasive investigations were performed. A 10-day scalp video-EEG monitoring revealed right temporal epileptiform discharges on interictal recordings and a right temporoparietal onset on ictal recordings of 6 habitual electroclinical seizures. Structural MRI showed multiple bilateral periventricular heterotopias, including nodules in temporal and occipital areas.

### 2:35 Findings on PET Scan and SISCOM.

PET scan revealed hypometabolism in the medial and lateral temporal regions and in the orbitofrontal cortex, while SISCOM showed hyperperfusion in the right frontal, temporal, and insular areas.

### 2:50 Magnetoencephalography Findings.

A magnetoencephalography was also performed. Most dipoles were localized to the right anterior and middle temporal lobe and in the orbitofrontal area.

### 3:02 EEG-fMRI Findings.

EEG-fMRI revealed activation in the right amygdala, frontal operculum, and anterior insula.

### 3:11 Resting-State fMRI Network Mapping.

Network mapping was also performed using resting-state fMRI. The network pattern included high connectivity between the right- and left-sided nodules and between ipsilateral nodules on the left.

### 3:27 Conclusion From Noninvasive Investigation Findings.

Taken together, semiology and noninvasive investigations were suggestive of a network characterized by a right occipital onset with rapid ipsilateral temporal spread. This being said, due to the presence of multiple nodules on MRI, an intracranial study was indicated.

### 3:48 SEEG Study.

A right-sided SEEG study was performed. Fifteen electrodes were implanted to cover the orbitofrontal area, the medial and lateral temporal regions, the insula, the nodules, and their overlying cortices.

### 4:06 SEEG Study Results.

Results of SEEG monitoring were suggestive of a complex epileptic network characterized by early ictal activity in a single right occipital nodule with rapid involvement of other nodules, followed by spread to the right mesiotemporal region. In addition, electrical stimulation of the right occipital nodule elicited habitual visual auras. These findings pointed to a key role for that heterotopia in the epileptic network.

Given the patient’s strong desire to drive, preservation of visual fields was paramount. Because open resection in the peri-atrial region is associated with high risk of injury to the optic radiations, a selective laser ablation was deemed the best option to avoid visual field impairments.

### 4:56 High-Resolution Tractography.

Preoperative planning included high-resolution tractography. Single-shell high angular resolution diffusion imaging incorporating 1 b0 image and 60 images with noncollinear diffusion gradients at b1500 was performed, and tracking was launched using the fiber orientation distribution function model to account for crossing and highly curved fibers such as those forming optic radiations. Preoperative tractography was crucial to determine the anatomical relationship between the targeted nodule (highlighted by the red arrow) and the adjacent optic radiations to be spared.

### 5:36 Trajectory Planning.

Prior to surgery, the desired ablation zone was delineated and the trajectory was planned through the occipital cortex along the long axis of the nodule.

### 5:47 Positioning and Bolt Insertion.

Following the placement of the CRW head ring and localizer frame, the patient underwent a head CT, which was then registered to the preoperative MRI. The patient was then placed in a semisitting position and the CRW head ring was attached to the operating table. The patient was prepped and draped in the usual fashion. The CRW Arc System was fixed to the head ring, and following a stab incision, a twist-drill burr hole was performed. The bolt was then inserted. Although the head was shaved for this particular patient, our more recent cases have been performed without shaving.

### 6:29 Video of the Thermal Ablation.

The patient was then transferred to the diagnostic MRI suite where the ablation was performed. Shown in white are the optic radiations reconstructed before surgery using high-resolution tractography and imported into the treatment software to aid in sparing these functional tracts.

A side-fire probe was inserted along the axis of the nodule to its deepest point. A distal-to-proximal ablation was then performed with the goal of ablating the entire nodule while avoiding injury to the optic radiations. To do so, the area of ablation was directed away from the optic radiations.

Thermal damage threshold lines are used to estimate the extent of ablation. The yellow lines correspond to reversible thermal damage, while the blue lines correspond to irreversible damage. These lines are monitored to limit the overlap with the optic radiations while optimizing ablation of the lesion. The objective is to have the entire heterotopia incorporated within the blue lines and to keep the optic radiations outside those lines as much as possible.

### 7:45 Overlap Between the Ablation and the Optic Radiations.

At the end of the ablation, minimal overlap was observed between the ablated area and the optic radiations.

### 7:55 Posttreatment MRI and Closure.

A posttreatment MRI, including contrast-enhanced T1-weighted sequences, was performed. The area of enhancement corresponds to the completed ablation. Final images revealed good overlay between the desired ablation planned before surgery (as shown by the green lines) and the enhancing ablated area.

The patient was then transferred back to the operating room, the bolt was removed, and the wound was closed with two staples.

### 8:24 Postoperative Course.

Following surgery, the patient was asymptomatic, with no neurological deficits on physical examination, and he was discharged home on postoperative day 1. At 3 months, a formal visual field test revealed no deficit. At the 12-month follow-up, the patient remained seizure free.

A prolonged EEG performed 6 months following surgery was significantly improved, revealing rare nocturnal interictal discharges. Despite the patient’s excellent seizure control, our plan is to continue long-term antiseizure medications to limit the risk of seizure recurrence.

### 9:02 Conclusion.

LITT is a safe alternative to open resection for periventricular nodular heterotopias surrounded by optic radiations.^9^ This approach allows to avoid direct transection of the optic radiations and injury to their arterial supply, two complications that are frequently observed following open transcortical resection.^9^

Advanced imaging such as high-resolution tractography may guide the ablation when incorporated into the treatment software. Tractography is therefore a useful surgical adjunct to preserve functional white matter tracts such as optic radiations.

As illustrated by our case, even in the presence of a complex epileptic network with multiple nodular heterotopias, LITT targeting a single nodule may be effective to control seizures in well-selected patients.

Finally, when managing complex epileptic networks, long-term treatment with antiseizure medications should be considered following surgery to avoid seizure recurrence.

## Disclosures

Dr. Weil reported personal fees from Monteris Medical outside the submitted work, and advisory board member for Synergia SA.

## Author Contributions

Primary surgeon: Weil, Bouthillier. Assistant surgeon: Obaid. Editing and drafting the video and abstract: Obaid, Guillemette, Lapalme-Remis. Critically revising the work: all authors. Reviewed submitted version of the work: all authors. Approved the final version of the work on behalf of all authors: Obaid. Supervision: Obaid, Bouthillier. MRI data and tractography processing: Yang.

## Supplemental Information

### Patient Informed Consent

The necessary patient informed consent was obtained in this study.
